# The RIDL hypothesis: transposable elements as functional domains of long noncoding RNAs

**DOI:** 10.1261/rna.044560.114

**Published:** 2014-07

**Authors:** Rory Johnson, Roderic Guigó

**Affiliations:** 1 Centre for Genomic Regulation (CRG), 08003 Barcelona, Spain; 2 Universitat Pompeu Fabra (UPF), 08003 Barcelona, Spain; 3 Institut Hospital del Mar d'Investigacions Mèdiques (IMIM), 08003 Barcelona, Spain

**Keywords:** long noncoding RNA, lncRNA, transposable element, transposon, repeat element, genome, evolution, functional domain

## Abstract

Our genome contains tens of thousands of long noncoding RNAs (lncRNAs), many of which are likely to have genetic regulatory functions. It has been proposed that lncRNA are organized into combinations of discrete functional domains, but the nature of these and their identification remain elusive. One class of sequence elements that is enriched in lncRNA is represented by transposable elements (TEs), repetitive mobile genetic sequences that have contributed widely to genome evolution through a process termed exaptation. Here, we link these two concepts by proposing that exonic TEs act as RNA domains that are essential for lncRNA function. We term such elements Repeat Insertion Domains of LncRNAs (RIDLs). A growing number of RIDLs have been experimentally defined, where TE-derived fragments of lncRNA act as RNA-, DNA-, and protein-binding domains. We propose that these reflect a more general phenomenon of exaptation during lncRNA evolution, where inserted TE sequences are repurposed as recognition sites for both protein and nucleic acids. We discuss a series of genomic screens that may be used in the future to systematically discover RIDLs. The RIDL hypothesis has the potential to explain how functional evolution can keep pace with the rapid gene evolution observed in lncRNA. More practically, TE maps may in the future be used to predict lncRNA function.

## Abstract (precis)

This paper discusses the provocative hypothesis that functional regions of long noncoding RNAs might be derived from sequences present in transposable elements.

## INTRODUCTION

One of the great surprises from the past decade of genomics has been the discovery of many thousands of long noncoding RNA (lncRNA) transcripts: The latest gene count in human has reached 13,000 (Gencode18) (Derrien et al. 2012); and with improving gene annotations, as well as rapidly increasing volumes of RNAseq data (Hangauer et al. 2013), it is likely that it will soon exceed that of protein coding genes. We do not yet know what proportion of lncRNAs in these annotations are true genes (Graur et al. 2013) and which are simply transcriptional noise (van Bakel et al. 2010). However, evolutionary evidence (Ponjavic et al. 2007) and a growing roster of experimentally demonstrated cases (Amaral et al. 2011) argue for a substantial core of bona fide genes that fulfill the strictest definitions of function. Based on a growing body of literature, lncRNAs would appear to primarily function as regulatory molecules both in the nucleus and cytoplasm through a wide repertoire of mechanisms, including interaction with epigenetic protein complexes (Rinn et al. 2007) and transcription factors (Kino et al. 2010), and hybridization to complementary RNA (Gong and Maquat 2011) or DNA sequences (Simon et al. 2011). This has opened new avenues in the study of human disease and biological processes (Faghihi et al. 2008; Gupta et al. 2010; Ng et al. 2012). Despite this progress, we still only have experimental information for about 130 or 1% of annotated lncRNAs (Amaral et al. 2011). In part, this is due to our lack of understanding of fundamental aspects of lncRNA biology, most notably the relationship between sequence and function, and our consequent inability to predict lncRNA function based on informatics analysis. To crack this sequence-function code, we must understand and categorize the active domains of lncRNA, what is their mechanism of action, and how they are combined to yield a functional molecule.

In this article, we propose that one of the keys to understanding RNA function lies in the transposable element (TE) sequences that they abundantly contain. Specifically, we will argue that TEs contribute preformed structural and sequence features that impart on lncRNA the ability to interact with and regulate other molecules. By rapidly and continuously shuffling such domains within new and existing lncRNAs, TEs have the potential to explain the evolution of complex lncRNA regulatory networks.

## THE CHALLENGE AND PROMISE OF MAPPING lncRNA FUNCTIONAL DOMAINS

It was recently proposed that lncRNA follow a modular organization, like proteins, composed of discrete domains that in combination determine the lncRNA's function (Guttman and Rinn 2012). This is an attractive hypothesis with various conceptual and practical implications. In evolutionary terms, domain organization explains how insertion or rearrangement of functional subunits can alter the function of existing genes or create novel ones relatively rapidly, through reuse of existing functional sequence rather than continual de novo evolution. Given that domains usually originate as duplications from a reduced number of canonical types related by structure and function (at least in proteins) (Koonin et al. 2002), we can identify them from primary sequence analysis, and classify them using sequence or structural similarity. Moreover, we may use this information to predict the function of novel genes by analysis of their primary sequence. Modular organization implies having distinct functionalities encoded by discrete sequence regions, separated by flexible linkers, and independent of context (Guttman and Rinn 2012). In lncRNA, functional domains are likely to act in at least two distinct ways: (1) adoption of a specific secondary structure that mediates the interaction with a protein partner; and (2) sequence-based hybridization to another nucleic acid. In this review, we use the term domain rather loosely, to include any clearly defined and self-contained region that confers upon its host transcript some biological activity, including functional structures or sequence motifs that interact with other molecules, but also regions that influence trafficking or processing, such as miRNA binding sites.

At present, our understanding of lncRNA domains and domain organization is limited to a small number of molecular biological and biochemical studies. These generally support the modular view, showing that lncRNAs are organized into discrete units at structural and functional levels, which retain their biological activity when separated from the rest of the molecule. An excellent case in point is represented by XIST, a 17-kb 8-exon transcript that is expressed from and represses one copy of the female X-chromosome in eutherians (Brown et al. 1991). A series of 7.5 repeats, termed A-repeats, are necessary for chromosomal silencing through recruitment of the PCR2 repressor complex (Zhao et al. 2008). Although the solution structure of the A-repeats has been the topic of debate (Wutz et al. 2002; Zhao et al. 2008; Maenner et al. 2010), the latest evidence suggests that the two halves of each repeat play distinct roles: The 5′ unit forms a highly stable hairpin structure, whereas the 3′ portions form intermolecular hybrids with their counterparts from the other repeats (Duszczyk et al. 2011). This silencing domain is distinct from localization activity, which is encoded by dispersed elements elsewhere in the transcript and which are unaffected by 5′ deletions (Wutz et al. 2002). One advantage of working with XIST is the possibility to do functional studies using cell lines overexpressing variants of an XIST transgene, where the impact of mutations on function is read out by measuring resultant changes in X-chromosome silencing and cell survival (Wutz et al. 2002). Such studies show that sequence mutations that do not alter the A-repeat structure have weak effects on function, whereas mutations affecting structure result in abrogation of XIST-mediated silencing (Duszczyk et al. 2011). This implies that, at least in the case of A-repeats, function depends in large part on RNA adopting the correct structure, regardless of sequence. Finally, the A-repeat region's function is independent of context, since a shorter XIST isoform, termed RepA, is also capable of interacting with PRC2 in vivo (Zhao et al. 2008).

Other functionally validated lncRNA also have modular organization. HOTAIR has two protein-binding domains at the 5′ and 3′ end that bring together two distinct repressor complexes, PRC2 and REST, respectively, at sites of gene repression (Tsai et al. 2010). Another HOX locus transcript, HOTTIP, recruits WDR5 chromatin remodeling protein through a domain at its 5′ end (Wang et al. 2011). The well-studied SRA coactivator transcript represents a case of structural modularity: Here the whole transcript would appear to be necessary for transcriptional activation, but the distinct structural subunits that contribute to this activity are themselves modular (Novikova et al. 2012). Thus, lncRNAs appear to be hubs where nucleic acids and proteins can be brought together, and it is precisely their domain structure that underlies this.

At present, we have no method of systematically identifying lncRNA functional domains. The development of such methods is hindered by a number of factors, most obviously the aforementioned small number of validated cases to be used as training sets. The ability to identify lncRNA domains would represent a major breakthrough because it would enable us to predict a priori the functions of the many thousands of lncRNA now known. In the case of proteins, this is now straightforward: Clearly identifiable primary, secondary, and tertiary sequences can be used to predict molecular activity and infer function, and such prediction for novel protein sequences is routine (Baker and Sali 2001). Although many methods exist for predicting RNA secondary structure with varying accuracy (Zuker 2003), we cannot presently link these to function.

Some progress has been made toward large-scale lncRNA functional prediction through a number of approaches. Recently, Glazko et al. (2012) trained a SVM predictor for lncRNA interactions with the Polycomb complex on human data, which seems to be effective in predicting mouse interaction data. The predictor was trained on the Khalil et al. (2009) PRC2 RIP-chip data and identified a combination of k-mers, TRANSFAC motifs, and sequence complexity that was enriched in the PRC2-binding RNAs compared to nonbinders. The method correctly identified known binders such as XIST and HOTAIR. However, it remains unclear how these classifiers relate to the true underlying mechanism of lncRNA-PRC2 recognition; and indeed, it remains formally possible that the classifier was identifying some other confounding aspect of lncRNA behavior, such as expression level, rather than specific PRC2 recognition.

Computational methods have been published for predicting protein-lncRNA interactions but these have not been extensively validated with high-throughput experimental data (Bellucci et al. 2011; Muppirala et al. 2011; Wang et al. 2013b). Encouragingly, methods developed recently, such as iCLiP and RIP-seq, are providing large-scale experimental maps of protein–RNA interactions, which include lncRNA and may offer clues to function (Yang et al. 2011). Similar to Glazko et al.'s (2012) results, these protein-binding data sets tend to identify short sequence motifs in binding sites. In light of their low specificity, it is not clear whether these motifs alone specify binding, or whether larger but cryptic sequence features also specify binding.

Although promising, the preceding methods do not yet yield large-scale information on lncRNA functional domains. Results from Glazko et al. (2012), as well as various iCLiP data sets, indicate that lncRNA molecular interactions are encoded in discrete sequence features that can be identified informatically, and these features are modular in the sense that they have similar functions in a wide number of lncRNA settings. One key feature of functional sequences is that they should be stereotypical—they should have similar sequence features in a large number of lncRNAs. We might take advantage of this observation to search for candidate functional elements by searching for overrepresented sequence features in lncRNA.

## TE SEQUENCES ARE ABUNDANTLY FOUND IN lncRNA EXONS

An obvious group of repeated sequence features within lncRNA are transposable elements (TEs). TEs are represented by various classes of repetitive, mobile sequence elements of varying origin and evolutionary age that constitute between one-half and two-thirds of our entire genomic sequence (Lander et al. 2001; de Koning et al. 2011). Previously regarded as purely parasitic elements, it is now broadly acknowledged that TEs play fundamental roles in cellular processes and in the evolution of genetic novelty (Cordaux and Batzer 2009). The evolutionary process by which TE sequences are subverted for novel function by the host genome is known as “exaptation” (de Souza et al. 2013). There is extensive literature demonstrating that TEs have contributed repeatedly and profoundly to the evolution of genome structure and function through the insertion of preformed sequence elements, both at the level of genomic DNA, e.g., transcription factor binding sites (Johnson et al. 2006), splice sites (Sela et al. 2010), enhancer elements (Huda et al. 2011b), and promoters (Huda et al. 2011a), and at the level of RNA, e.g., microRNA genes (Spengler et al. 2014), recognition elements (Piriyapongsa and Jordan 2007), and protein-coding domains (Bowen and Jordan 2007).

**FIGURE 1.**
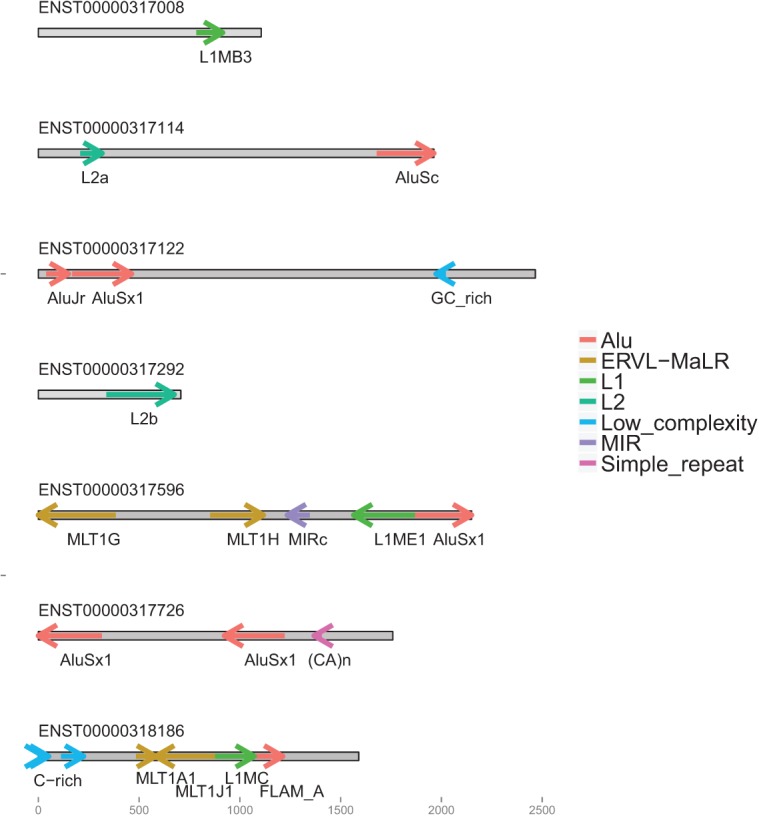
Examples of TE insertion profiles in annotated lncRNA. Insertions are represented by arrows, colored by TE class. The rectangles represent mature lncRNA transcripts and are to scale.

Recently, a number of studies have highlighted an intriguing relationship between TE sequences and long noncoding RNA. A large proportion of exonic lncRNA sequence has originated from TEs: Based on a mixed lncRNA annotation from RNA sequencing and GENCODE, Kelley and Rinn (2012) estimated that 41% of lncRNA nucleotides are derived from TEs, and the majority of lncRNAs (83%) contain at least one TE fragment. As a consequence, many mature lncRNA transcripts contain combinations of multiple repeat fragments reminiscent of protein domain structures (Fig. 1).

Particular families of TEs are strongly and nonrandomly enriched or depleted from lncRNA sequence: Kelley and Rinn (2012) found a particularly strong overrepresentation of human endogenous retrovirus (hERV) families in lncRNA exons compared to the genomic background, but other classes such as LTR subtypes and MLT are also enriched. In contrast, families including the highly numerous Alu, L1, and L2 classes are significantly depleted from lncRNA. These patterns suggest that the presence of TE fragments within mature lncRNA sequence might have been selected for or against during evolution.

TEs have had a profound influence on lncRNA gene structure, particularly in terms of regulatory regions and splice sites. In another recent paper, Cedric Feschotte and colleagues found numerous examples in which lncRNA promoters, splice donor, and splice acceptor and polyadenylation sites are composed of TE-derived sequence (Kapusta et al. 2013), echoing a previous study demonstrating widespread alternative promoter contributions by TEs (Faulkner et al. 2009). The TE content of lncRNA genes far exceeds that of protein-coding genes, almost certainly due to the inability of protein-coding sequence to tolerate insertions (Sela et al. 2010). Kelley and Rinn (2012) went further to show that the 127 lncRNAs promoted by HERVH elements are specifically up-regulated in pluripotent cell types (Kelley and Rinn 2012), which is consistent with previous observations of the overexpression of these elements in human embryonic stem cells (Santoni et al. 2012). Indeed it is likely that TEs such as HERVH are actually responsible for the birth of new lncRNAs by the insertion active promoters into previously inactive genomic regions (Kelley and Rinn 2012). It is worth noting that HERVH is among the most enriched elements in lncRNA exonic sequence. Thus, TEs contain preformed sequence motifs that have driven the evolution of lncRNA gene structures and indeed to the evolution of new lncRNAs.

The presence of TE sequence within a lncRNA does not appear to be detrimental, and clear cases exist of repeat-rich, functional lncRNAs. The transcript linc-RoR, identified in pluripotent embryonic stem cells, is capable of increasing ESC reprogramming efficiency when included with the standard Yamanaka factors (Loewer et al. 2010). The mature linc-RoR transcript is composed of ∼70% TE-derived sequence from multiple families. Although the location of this functionality within linc-RoR has not been mapped, the extent of repetitive sequence in the transcript, as well as the observed link between TEs and pluripotency, is highly suggestive of a role of endogenous retroviral sequence in promoting pluripotency (Santoni et al. 2012).

Several other repeat-rich lncRNAs have been described. In mouse, a brain-specific transcript AK046052, regulated by the master neural transcriptional repressor REST, is largely a mosaic of TE-derived sequence (Johnson et al. 2009). Kelley and Rinn (2012) also highlighted a number of functionally characterized lncRNAs, such as TUG1 (Young et al. 2005) and BANCR (Flockhart et al. 2012), that contain significant amounts of TE-derived sequence. Perhaps the most compelling example comes again from XIST, whose TE content has actually increased in the human lineage since its evolutionary repurposing from a protein-coding gene (Elisaphenko et al. 2008). Overall, we might conclude that TE sequence within lncRNA is the rule rather than the exception, and high levels of TE insertion are compatible lncRNA activity.

What is less clear from these studies, however, is to what extent TEs have contributed to functional sequence within the lncRNA transcript itself. Indeed, given cases like linc-RoR, it is possible that, far from impairing function, TEs are necessary for lncRNAs molecular activity. The enrichment (and indeed depletion) of particular TEs would appear to argue that they have been selected for or against within lncRNAs, and thus, their presence has directly contributed lncRNA function.

## HYPOTHESIS: TRANSPOSABLE ELEMENTS AS FUNCTIONAL DOMAINS OF lncRNAs

The abundant and nonrandom insertion of TE into lncRNA exons reviewed above leads us to propose the following related hypotheses:

The set of TE insertions within lncRNA exons contains a subset of biologically active sequences that are important for lncRNA function; and
TE insertion is a general evolutionary mechanism by which lncRNA functionality evolves through the combinatorial addition of distinct TE domains that result in emergent and complex properties in their host lncRNA.

Together these hypotheses can help to explain one of the outstanding questions regarding lncRNAs: How can these genes, which are born over relatively short evolutionary timescales, rapidly acquire molecular activity and play new functional roles? A newly expressed, nonfunctional lncRNA may transcribe a preexisting TE fragment. Alternatively, a TE may be inserted within an existing, functional lncRNA. In either case, if the TE sequence in question has some kind of biological activity, it may confer that activity on the host lncRNA and at a small but definite frequency confer a selective advantage.

**FIGURE 2.**
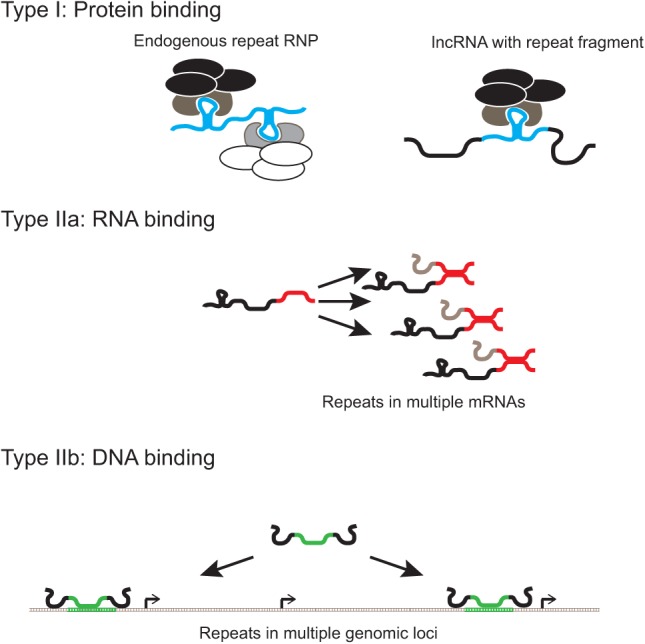
Functional classification of exonic TE insertions: (black) lncRNAs; (ovals) proteins; (gray) interacting mRNAs/lncRNAs/genomic DNA; (arrows) gene promoters.

How could TE-derived sequences contribute to lncRNA functionality? We next consider two principle alternatives (Fig. 2). First, within the lncRNA, the TE sequence continues to perform a similar function as that for which it evolved in the ancestral TE, most likely through protein binding (Blackwell et al. 2012). Alternatively the TE sequence might mediate hybridization to other, homologous nucleic acid sequences (Gong and Maquat 2011). In summary, we propose two principle classes of functional TE sequence within lncRNA (Fig. 2).

### Type I: protein interaction

In the course of their normal cellular lifecycle, TE transcripts interact with a variety of proteins, both self-encoded and host-encoded, to form a ribonucleoprotein complex (RNP) (Goodier et al. 2013). RNPs, such as Alu or LINE1, have been shown to interact with a diverse range of host proteins, including chromatin modifiers, transcription factors, DNA repair factors, RNA binding proteins, and RNA Polymerase II (Mariner et al. 2008; Blackwell et al. 2012; Goodier et al. 2013). It is reasonable to infer that fresh insertion of TE repeats within lncRNA may confer binding to the same complexes, thereby constituting preformed protein-binding domains. Among those protein classes recently found to interact with Alu and LINE1 are many, such as chromatin regulatory complexes, that are highly relevant to known functional roles of lncRNA (Blackwell et al. 2012; Goodier et al. 2013). Thus, there is a relationship, at least at early evolutionary stages, between the TE's activity in the lncRNA context and its role in its original TE context.

### Type II: nucleic acid interaction

Repeat elements might also confer functionality through their sequence alone, and its ability to specifically hybridize to the multiple other copies of the same repeat element that exist, by definition, throughout the genome. In contrast to Type I, the functionality of such sequence is not necessarily related to its functionality (if any) within the endogenous TE. The specificity of this interaction will depend both on the length of the TE fragments, as well as their originating from the same fragment of the TE consensus sequence. Such hybridization may occur through Watson-Crick base-pairing by the lncRNA-embedded repeat to either DNA or RNA sequences (Gong and Maquat 2011):

**1. Type IIa: RNA binding**
Inserted TE could confer sequence-specific RNA-binding modules through simple complementarity. The advantage of this is that such binding would occur in sequences derived from the same or related TE families on the opposite strand of the target molecule, thus enabling the evolution of a large repertoire of highly similar target sequences of extended length, and hence specificity (Fig. 2). An example of this is targeting of mRNAs for Staufen-mediated decay by lncRNA through Alu-mediated complementary base-pairing (Gong and Maquat 2011).

**1. Type IIb: DNA binding**
It is likely that lncRNA are capable of interacting directly with genomic DNA sequence through conventional Watson-Crick base-pairing or through alternative modes such as Hoogsteen base-pairing (Buske et al. 2012). As in the aforementioned case of RNA, the abundance of near identical TE elements within genomic DNA offers a plausible model whereby complementary interactions mediated by embedded TE sequences with DNA could target lncRNAs to specific genomic loci (Fig. 2). This model has been proposed for Alu fragments within the *ANRIL* lncRNA (Holdt et al. 2013).

**FIGURE 3.**
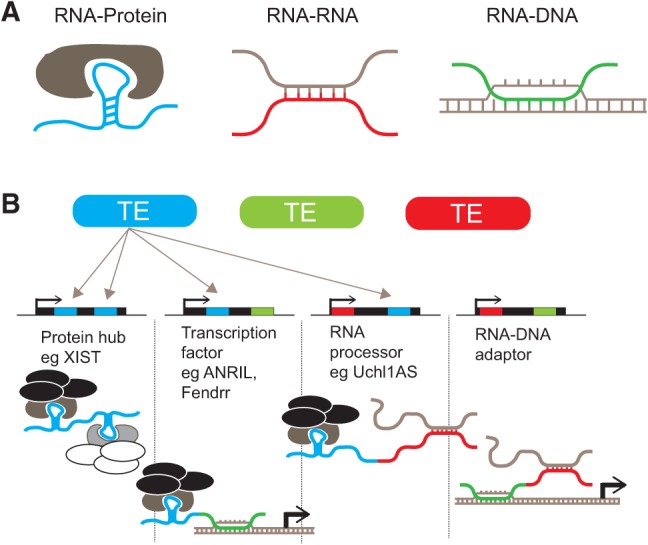
(*A*) Activities of distinct RIDLs. (*B*) Evolution of diverse lncRNA functions through TE integration.

The precise functionality of lncRNA, like protein, resides in the combinatorics of its constituent functional domains. In other words, different combinations of the TE-derived domains mentioned above could give rise to lncRNAs with different regulatory abilities (Fig. 3). For example, multiple distinct protein-binding sites would function to unite proteins or protein complexes (such as HOTAIR) (Tsai et al. 2010). The combination of RNA binding domain with protein binding could give rise to a regulator of mRNA processing, represented by *Uchl1as* for example, whose antisense domain specifically targets *Uchl1* mRNA, while its SINEB2 repeat potentiates translation (Carrieri et al. 2012). Finally, we propose a hypothetical RNA–DNA adaptor configuration that might serve to recruit other ncRNAs (or even mRNAs) to specific genomic locations (Fig. 3).

The combination of a DNA-binding sequence with a protein-binding domain might give rise to a “transcription factor” lncRNA that recruits gene regulatory or epigenetic complexes to defined genomic regions (for example, *Fendrr*) (Grote et al. 2013). Presumably, this is how HOTAIR functions, since it is known to interact with the chromatin regulatory complexes, such as PRC2 and REST, and its binding sites as mapped by CHIRP (Chu et al. 2011) contain an enriched GA-rich sequence motif that might be recognized by HOTAIR itself (although it has not been definitively resolved whether HOTAIR directly binds to this motif, or how). Here we have discussed the simplest two-domain combinations, but an essentially infinite variety of possible combinations between nucleic-acid and protein-binding domains exist.

Within lncRNAs, these TE-derived domains would be expected to be interspersed with poorly conserved linker regions, as proposed by Guttman and Rinn (2012). Furthermore, one might expect that the extensive alternative splicing witnessed in lncRNA genes (Derrien et al. 2012) might give rise to transcripts with various combinations of protein- and nucleic acid-binding domains.

In the following sections, we discuss first the experimental evidence supporting this hypothesis, the implications for our understanding of lncRNA evolution, and finally, some methods for the systematic discovery of TE-derived lncRNA functional domains.

## TRANSPOSABLE ELEMENT RNA IS BIOLOGICALLY ACTIVE

There is a growing body of experimental literature that supports the idea that TE fragments within lncRNA contribute to function. These include cases, discussed below, in which TEs have clear, RNA-based biological activity either in isolation (this section), within the context of another RNA molecule (principally mRNAs), or direct evidence of functional TEs within lncRNA (next section). In this section, we discuss the former case, in which there is evidence for intrinsic biological activity for natural TE RNA sequence. These cases have particular relevance where we propose that host lncRNAs acquire aspects of the original activity of their TE repeats.

TE transcripts have been shown to have activity at the whole-cell level as well as in human diseases and at the molecular level. In addition to being activity transcribed in cell compartment (Goodier et al. 2010), developmental (Rowe and Trono 2011), and tissue-specific patterns (Faulkner et al. 2009), many TE insertions are under purifying evolutionary selection (Lowe et al. 2007). There is evidence for biological activity of repeats from a range of classes, from the large, autonomous long interspersed nuclear elements (LINEs), through various virally derived long terminal repeat (LTR) sequences, and to the nonautonomous short interspersed nuclear elements (SINEs).

There is a range of evidence attesting to the activity of Alu sequence at both the DNA and RNA levels in both healthy and diseased tissues. This highly numerous, short (300 nt), structured element is derived from the 7SL signal recognition particle RNA and has expanded massively in the primate lineage (Lander et al. 2001; Giordano et al. 2007; Mariner et al. 2008). It was recently shown that age-related macular degeneration arises from aberrant Dicer processing in the retina, leading to the accumulation of Alu transcripts, which results in toxicity and consequently retinal neuronal degeneration (Kaneko et al. 2011). A recent screen for binding partners of Alu sequence discovered a diverse repertoire of protein partners, including a number of chromatin remodeling factors and transcription factors (Blackwell et al. 2012). Indeed, ongoing work by Kugel and Goodrich have demonstrated that Alu and other SINE transcripts are capable of binding and repressing RNA Polymerase II activity through the adoption of a modular structure, thereby repressing global gene transcription during heat shock (Mariner et al. 2008). These data suggest that Alu transcripts may directly participate in genomic regulatory processes through protein interactions.

Alu are not alone in their abundant expression and clear phenotypic effects on their host cells. A recent study also found that L1b retrotransposons are associated with the chromatin modifying complexes that maintain neocentromeres (Chueh et al. 2009). More evidence for binding to protein complexes comes from a recent analysis of TDP43, the RNA binding protein involved in multiple neurodegenerative conditions (Li et al. 2012). Here, the authors showed that TDP43 is bound by a wide variety of TEs in both human and mouse neural cells, and this association is disrupted in disease, raising the possibility that differential protein binding by TE transcripts may play a role in neurodegenerative processes. Finally, we recently showed that transposable elements are globally derepressed in cancer, suggesting that their expression contributes to malignancy (Ferreira et al. 2014), possibly by inserting and altering transcription of proto-oncogenes or tumor suppressors (Shukla et al. 2013).

TE transcription appears to be a normal and regulated process during development. In mouse preimplantation blastocysts, LTR-type transposons are actively transcribed and contribute many cell-stage-specific promoters to other genes (Peaston et al. 2004), reminiscent of ESC-specific expression of HERVH-driven promoters (Kelley and Rinn 2012). In undifferentiated neural precursor cells of human and mouse, LINE1 elements are globally derepressed, resulting in cell-specific insertion events and genetic mosaicism in adult neurons (Muotri et al. 2005; Baillie et al. 2011).

Although far from conclusive, these data together suggest that TE RNAs may play causative roles in fundamental cellular processes. Furthermore TEs have many of the hallmarks of functional ncRNAs: modular structural organization, protein interaction, specific trafficking within the cell, and evolutionary conservation.

## DIRECT EVIDENCE FOR TE-DERIVED FUNCTIONAL DOMAINS IN lncRNAs

The hypothesis that TE sequences can act as functional domains of lncRNA has recently gained support from a number of experimental studies, which provide examples for all but one of the scenarios outlined in Figures 2 and 3. These cases are discussed below, and summarized in Table 1.

**TABLE 1.**
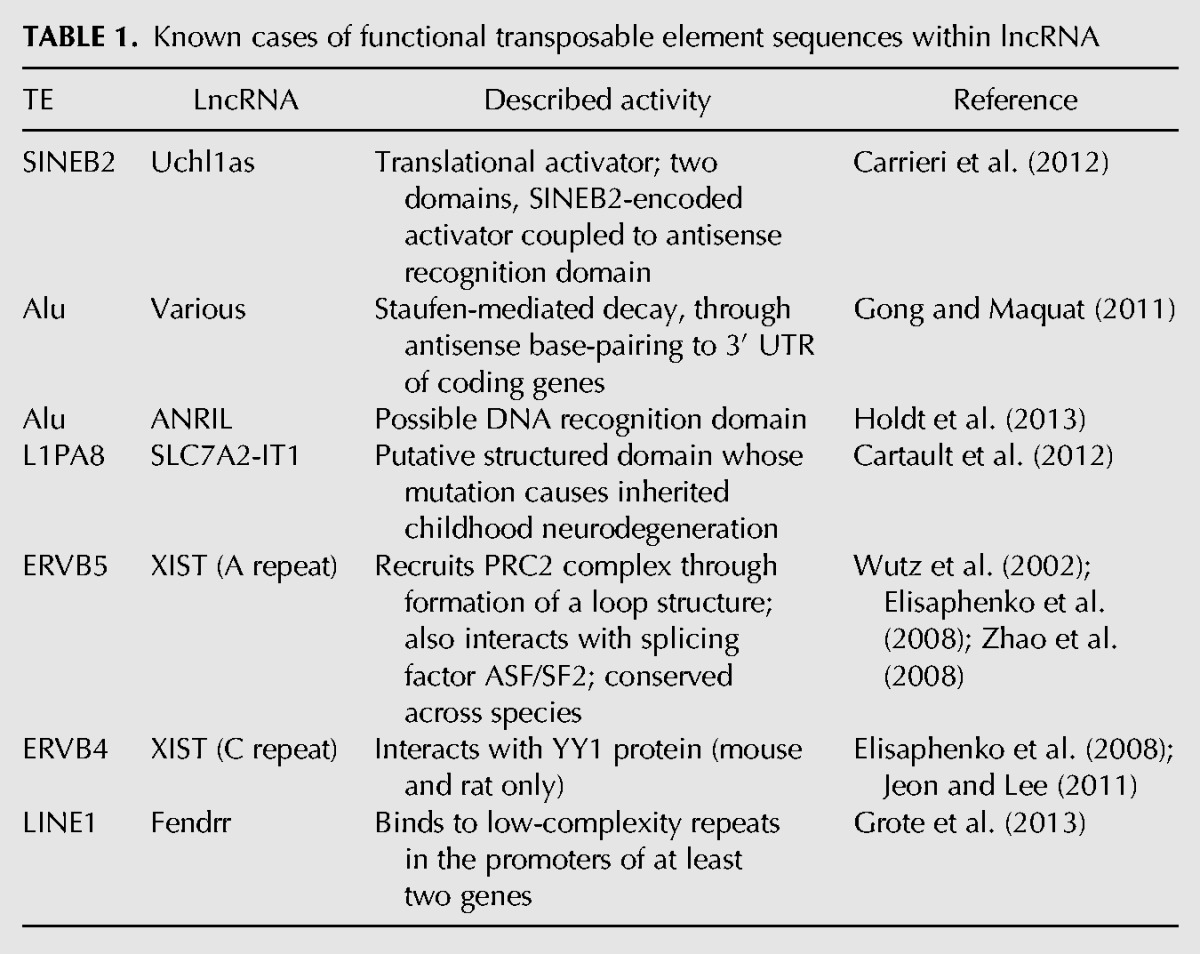
Known cases of functional transposable element sequences within lncRNA

We commence with the longest studied and most clearly functionalized lncRNA, XIST, whose key role in mammalian genetics is underwritten by at least three distinct, repeat-derived functional domains. Silencing by XIST strictly depends on the presence of the 5′ repetitive A-repeat domain, which is conserved across eutherians (Wutz et al. 2002; Elisaphenko et al. 2008). Deletion of this region ablates the repressive function of XIST, while leaving its targeting largely unaffected (Wutz et al. 2002), although A-region mutants do appear to have deficiency in crossing active chromosomal regions (Engreitz et al. 2013). The A-repeat region, as mentioned above, adopts a structural configuration that interacts with the repressive PRC2 complex to repress chromatin (Zhao et al. 2008). The origin of the A-repeat region was recently shown to have most likely originated as an endogenous retrovirus, ERVB5 (Elisaphenko et al. 2008). In contrast, the localization of XIST seems to be dependent on sequences more dispersed throughout the transcript (Wutz et al. 2002), although a later study using targeting antisense oligonucleotides implicated the murine-specific C-repeat region in correct targeting through unknown mechanisms (Beletskii et al. 2001). This targeting is mediated by the specific interaction of repeat C with the transcription factor, YY1, that directs XIST to specific genomic loci through DNA binding (Jeon and Lee 2011). This region also has a repetitive origin, having homology to another endogenous retrovirus, ERVB4 (Elisaphenko et al. 2008). Most recently, it was shown that the conserved Repeat F is part of the core region necessary for Jarid2 interaction and may have originated from a DNA transposon (Elisaphenko et al. 2008; da Rocha et al. 2014). Thus, the distinct functionalities of XIST, targeting and silencing, appear to have evolved from transposable elements, which in combination give XIST at least three distinct protein-binding modules as depicted in Figure 3B.

**FIGURE 4.**
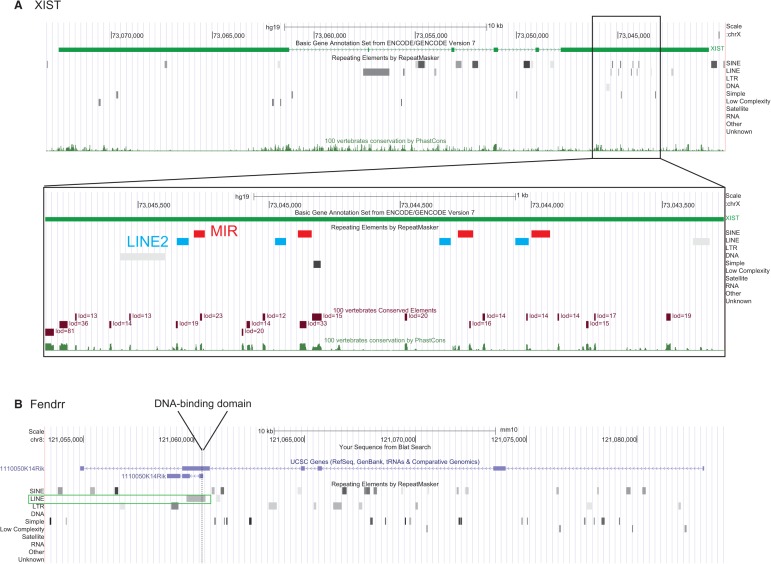
(*A*) The human XIST locus, included a zoomed-in region of exon 6 showing the mentioned LINE2 and MIR repeats. Note their intersection with PhastCons conserved elements (dark red, *lower* track). (*B*) The mouse Fendrr locus, indicating the experimentally mapped DNA-binding domain (Grote et al. 2013) and its overlap with an Lx8 LINE1 insertion (green box).

One intriguing observation is the long acknowledged correlation between X-chromosome gene targeting by XIST and the density of TEs around the promoters of those genes (Wang et al. 2006). It is unclear whether the repeat content of XIST is in any way related to the unexplained relationship between the efficiency of silencing of genes on the X-chromosome and the distribution of repeat elements in their genomic neighborhood. Recent, sequencing-based maps of XIST along the inactive X have revealed a number of such relationships, both positive and negative, at unparalleled resolution (Engreitz et al. 2013). Strikingly, in both human and mouse, the genes on the X-chromosome silenced by XIST are significantly and positively correlated to the density of MIR and L2 elements around their promoters (Wang et al. 2006; Engreitz et al. 2013). Inspection of the last exon of XIST shows four sets of LINE2 and MIR repeats, with conserved orientation that presumably have resulted from two rounds of sequence duplication (Fig. 4A). These repeats in several cases overlap regions of elevated vertebrate sequence constraint. Together these observations lead us to speculate that these LINE2-MIR subunits contribute to the targeting of XIST to the promoters of silenced target genes on the X-chromosome through Watson-Crick base-pairing. Future studies will be required to test this hypothesis. TEs can also contribute DNA-binding domains to lncRNA (Type IIb in Fig. 2): A recent study of the coronary artery disease-associated lncRNA, ANRIL, showed that Alu elements within its sequence were necessary for its biological activity; and loss of embedded Alu elements reversed ANRIL's promotion of growth, adhesion, and motility in cell models (Holdt et al. 2013). ANRIL binds to various epigenetic regulatory proteins, including members of the PRC1 and 2 complexes; intriguingly, Alu sequences complementary to those in ANRIL tend to have very specific spacing relative to PRC binding sites. Although the implications remain unclear, we speculate that Alu motifs target the ANRIL-PRC2 complex to complementary genomic sites.

Another TE-derived DNA-binding domain has been identified in the mouse lncRNA, *Fendrr*, which is necessary for mouse heart and body wall development (Grote and Herrmann 2013). The authors showed evidence that *Fendrr* directly binds to at least two gene promoters to which it recruits various chromatin-remodeling factors. Inspection of the putative DNA-binding domain of *Fendrr* shows that it is derived from a LINE1 element (Fig. 4B). Thus, ANRIL and *Fendrr* constitute two examples in which TE-derived fragments mediate the lncRNA genomic targeting by Watson-Crick base-pairing, corresponding to the “transcription factor” model shown in Figure 3B.

The idea of TEs contributing RNA-binding domains to lncRNA (Type IIa) also has been experimentally validated. Lynne Maquat's laboratory has shown in a series of papers that mRNAs are targeted for degradation by the Staufen protein through Alu repeats in their 3′ UTR region (Gong and Maquat 2011). The recognition by Staufen requires the formation of a double-stranded RNA substrate, originally identified through intramolecular base-pairing (Kim et al. 2007). Subsequently, they showed that Staufen targets may also form when mRNAs hybridize to lncRNA through complementary Alu fragments (Gong and Maquat 2011). A given lncRNA can target multiple different mRNAs through shared Alu sequences, providing an attractive model for post-transcriptional gene regulation by lncRNA, with specificity provided by TEs.

Further attesting to the significance of TE-derived lncRNA comes from an intriguing recent study on a rare neurodegenerative condition, infantile encephalopathy, which is restricted to a small population from the island of Reunion (Cartault et al. 2012). By genetic mapping, a single-nucleotide disease-causing mutation was discovered in a L1PA8 element embedded within a novel intergenic lncRNA locus with brain specific expression, SLC7A2-IT1. siRNA-mediated knockdown of SLC7A2-IT1 induced apoptosis in cultured neuroblastoma cells, suggesting that its expression is necessary for neuronal survival. The disease-causing mutation is predicted to fall within a structured region formed by the repeat element. The single postmortem brain sample the authors tested had strongly reduced levels of the host RNA, but brain-expressed protein coding genes located proximally to SLC7A2-IT1 in the genome were unaffected, suggesting that (1) the neurodegenerative phenotype is due to reduced levels of SLC7A2-IT1; (2) the L1 element somehow controls lncRNA steady state levels; and (3) that the lncRNA functions in *trans*. Another interpretation is that the L1 element serves to regulate transcription of lncRNA at the DNA level, and this hypothesis will have to be ruled out before we can definitively state that SLC7A2-IT1 represents a TE-derived lncRNA domain.

Finally, TEs have recently been shown to play an integral role in gene regulation by antisense lncRNAs. In a study on regulation of the neuronal-specific *Uchl1* mRNA by antisense transcripts, the authors unveiled an elegant principle of translational regulation: A bipartite antisense contains (1) a “targeting” module, antisense to its target mRNA, with (2) a downstream embedded SINEB2 repeat (Carrieri et al. 2012). The antisense hybridizes to the mRNA, whereas the SINE2B repeat up-regulates its translation through a mechanism that remains unclear. Removal of the SINE2B element completely abrogated the translational effect of the transcript. The authors found other similar examples and indeed were able to engineer synthetic lncRNAs to activate translation of a GFP transgene. It is likely that other antisense lncRNAs also bind their sense, coding transcript to effect other regulatory outcomes: BACE1-as binds to and increases the stability of BACE1 mRNA (Faghihi et al. 2008), whereas another neural antisense transcript, BDNFOS, negatively regulates BDNF mRNA (Lipovich et al. 2012). Both of these transcripts contain multiple exonic TE insertions, although these have not yet been strictly linked to function.

Together these cases provide a diverse body of evidence that TE-derived fragments can and do contribute nucleic acid and protein-binding modules that are strictly necessary for lncRNA's biological activity.

## TEs AND THE EVOLUTION OF COMPLEX lncRNA REGULATORY NETWORKS

One key biological challenge is to understand the genomic processes that underlie evolutionary changes, both in general and specifically between *Homo sapiens* and other primates. It has been proposed that lncRNA have played an essential role in the evolution of developmental gene regulatory networks underlying such changes (Britten and Davidson 1971; Pollard et al. 2006; Mattick 2009). Recent evidence would indeed support a widespread role for lncRNA in the regulation of key processes known to have undergone substantial evolutionary change between mammals, including stem cell pluripotency (Guttman et al. 2011), neurodevelopment (Ng et al. 2012), and immune function (Carpenter et al. 2013). Although recent studies have addressed the evolution of lncRNA genes (Necsulea et al. 2014; Washietl et al. 2014), the processes governing their functional evolution have not been investigated. Transposable elements are likely to have contributed to both processes.

LncRNA have several features distinct from proteins that would appear to give them an advantage as gene regulators in higher organisms:

They do not need to be translated into protein outside the nucleus, so that they become functional immediately upon transcription, and can regulate gene expression directly at their site of transcription (in *cis*), in addition to *trans* targeting.
They are intrinsically versatile in their molecular interactions: They can interact with other molecules through both structural and sequence-specific modes, giving them potential to bridge proteins and nucleic acids.
They are evolutionarily malleable, since their sequence can tolerate insertions or deletions, in contrast to protein-coding open reading frame sequences that in most cases cannot tolerate such mutations without a loss of function.

A regulatory role for lncRNA is supported by a wide range of observations: A large number are associated with epigenetic regulatory proteins (Khalil et al. 2009), they tend to be localized in the nucleus and chromatin (Clark et al. 2012; Derrien et al. 2012), although many are present also in the cytoplasm (Gong and Maquat 2011; Carrieri et al. 2012), and indeed a growing number of examples attest to their regulation of gene expression in both *cis* and *trans* (Gupta et al. 2010; Maamar et al. 2013). The exaptation of TE-derived modules in lncRNA is consistent with such a regulatory role, since such modules are likely to be capable of interacting with highly relevant regulatory protein complexes (e.g., Alu and chromatin regulatory factors), or by specific recognition of genes at both transcriptional (i.e., DNA recognition, such as ANRIL) or post-transcriptional stages (i.e., RNA recognition, such as the Alu-Staufen pathway).

To the preceding features we may also add a more general property of regulatory biomolecules, which is modularity. As discussed above, this composition of clearly defined subunits of distinct function is fundamental for two reasons: (1) It facilitates evolutionary innovation through the simple rearrangement or addition of domains within existing or new genes; and (2) modularity is required for the emergence of complexity in regulatory networks, since each domain represents a molecular interaction in a genetic pathway, and thus combinations of domains represent connections between such pathways. Such organization is ubiquitous in regulatory proteins; for example, a typical regulatory transcription factor will combine a DNA-binding domain, a protein-binding effector domain (often interacting with a chromatin modifying complex), and often some kind of sensor (for example, the ligand-binding domain, in the case of nuclear hormone receptors) (York and O'Malley 2010). The activity of the protein is determined by its domain structure, and this structure has been repeatedly shuffled through evolution to create new variation with altered functionality. One might imagine that by simply reshuffling combinations of genomic-targeting domains/RNA-targeting domains/activating or repressing domains, evolution could rapidly give rise to novel lncRNAs that connect different components of cellular networks.

In proteins, evolutionary tinkering in the form of domain shuffling takes place through insertion of novel coding sequences by a variety of genomic recombination mechanisms (Buljan et al. 2010). This process is strictly limited by the requirement that the newly inserted exon be in the same open reading frame as the host gene, limiting the frequency with which such events give rise to a viable protein. In the case of an inserted internal exon, for example, just one in three insertions will result in a viable protein (Marsh and Teichmann 2010; Schad et al. 2013). Similarly, although TEs have been shown to occasionally contribute novel exonic sequence to protein-coding genes, the insertion of a TE within a coding exon, or else the spliced inclusion of an entire TE-derived exon, only has a one-in-three probability of creating a viable protein, and even then it would likely be a stretch of nonsense protein (Sela et al. 2010). In contrast, lncRNA would be expected to accept TE sequence much more readily without adversely affecting their function since the RNA sequence function is not dependent on a strict frame or register. Indeed, it has been proposed that lncRNAs consist of small islands of functional sequence within large stretches of functionally and evolutionarily neutral sequence (Guttman and Rinn 2012). Therefore, lncRNA genes in general are more likely to accept new sequence contributions while maintaining functionality. This is reflected in the vastly higher rate of exonization of TEs in lncRNA compared to protein coding genes (Kapusta et al. 2013).

Transposable elements are highly clade specific, a fine example being the Alu element, which has expanded massively in the primate lineage (Giordano et al. 2007). A consequence of this is that TE activity might insert lineage-specific functional domains into a conserved lncRNA transcript, as suggested by Kapusta et al. (2013). This is an attractive mechanism to explain lineage-specific changes in gene networks controlled by lncRNA. This is particularly relevant given the described functional roles played by lncRNA-embedded Alus, including DNA binding (Holdt et al. 2013) and mRNA recognition (Gong and Maquat 2011). Interestingly, in the latter case, an analogous system evolved in the mouse lineage (which lacks Alu), where Staufen-mediated decay is instead mediated by recognition of other short repeat elements, the Mus-specific B1, B2, and B4 (Wang et al. 2013a). Another similar case of analogous RNA function again involves Alu in human and B2 in mouse, where both are capable of binding and repressing RNA Pol II (Yakovchuk et al. 2009). From these findings we might draw two conclusions: (1) Analogous evolution of TE function might take place in lncRNA from different evolutionary branches; and (2) TE activity may contribute to lncRNA evolution and divergence in particular lineages (similar to that observed for TE-driven transcriptional network rewiring) (Bourque et al. 2008).

An excellent example of lineage-specific TE insertion and acquisition of function was recently described for ANRIL (He et al. 2013). The evolutionary history of ANRIL in eutherians has been complex, apparently gaining exons in primates and most other lineages, but shrinking in rodents. In simians, a particularly complex gene emerged, and this process was accompanied by the fixing of multiple exonic TE insertions. In primates, ANRIL exons have come under selection following insertion of TEs. More intriguingly, those same exonic TE fragments have also experienced selection following insertion. Together, these data point to a situation in which a preexisting lncRNA acquired new functional domains through the TE insertions.

One key feature of TEs as targeting sequences is that, by their nature, they are highly abundant in the genome (for example, >1 × 10^6^ Alus; >0.5 × 106 LINEs) (Cordaux and Batzer 2009). Thus, any RIDL that operates through base-pairing to complementary nucleotide sequence, be it DNA or RNA, will have a multitude of potential binding sites throughout the genome. Not only are these sites abundant, but they are also highly specific, consisting of highly complementary fragments often >100 nt long and potentially participating in specific and energetically favorable binding. This specificity would appear to be a key advantage of lncRNA as a regulatory molecule compared to protein-based transcription factor, whose genomic binding motifs are unrelated to the encoded gene itself.

The processes by which lncRNAs are born is presently a focus of research (Necsulea et al. 2014; Washietl et al. 2014). Although outside the scope of this review, it is also worth mentioning that, in addition to contributing functional sequence to existing lncRNAs, TEs are also likely to be a driver in the birth of new lncRNA genes. This occurs through the insertion of novel TE promoter fragments in previously inactive genomic regions, driving the transcription of lncRNA transcripts that eventually acquire function. Kelley and Rinn (2012) showed at least one excellent example of this in which hERV-derived promoters drive the expression of a subset of lncRNAs specifically in pluripotent cells (Kelley and Rinn 2012). It will be fascinating to find out whether other classes of repeat drive lncRNA expression in other tissue types, and whether this mechanism is the principle driver of new lncRNA gene birth. It is also worth mentioning that such transcripts will necessarily carry some TE sequence at their 5′ end, which could conceivably contain functional elements.

We might consider two distinct functional roles of exapted TEs that will result in different distinct evolutionary patterns: function through structure (Type I) or function through sequence (Type II) (Fig. 2). In the case in which this involves the adoption of a structure for protein binding, then we might expect that the TE fragment will confer binding of the host lncRNA to natural partners of the TE, specifically the TE RNP (Fig. 2; Blackwell et al. 2012; Goodier et al. 2013). Such RNPs are known to interact with a wide range of proteins, including those with regulatory functions of clear relevance to lncRNA function (Blackwell et al. 2012). Thus, TE protein partners represent obvious candidates to interact with TE-containing lncRNAs. For the TE-derived fragments of this type, we would expect them to undergo purifying selection on RNA structure, with characteristic compensatory mutations (Smith et al. 2013), exactly as has been observed for the XIST A-repeats (Duszczyk et al. 2011).

On the other hand, exapted exonic TEs might function purely at the sequence level through hybridization to complementary sequences in DNA or RNA. In this case, we would expect evolutionary constraint on RNA sequence but not necessarily on structure. More specifically, we would expect constraint at the complementary sites to which the RNA is binding, meaning that there should be correspondence in the precise subregion of the repeat consensus found in the RNA and in its genomic binding site. Widespread conservation of intergenic TE fragments has already been observed (Lowe et al. 2007). Therefore, these differing constraints on exapted TE sequence may enable us to distinguish Type I and Type II domains (see below).

Here, we have speculated on the possible role that TEs have played in the evolution of regulatory lncRNAs. We conclude that the RIDL hypothesis of lncRNA evolution through acquisition of TE-derived functional domains is consistent with the observed rapid evolution of regulatory lncRNA. In the following section, we propose how we might go about systematically identifying exapted TE domains using various genomic analysis, including exploiting characteristic evolutionary patterns that such TE fragments might undergo.

## HOW TO FIND FUNCTIONAL TE DOMAINS GENOME-WIDE

The hypothesis that TEs have extensively contributed to lncRNA functional domains results in a number of testable predictions about their sequence characteristics that might be used to discover such exapted TE domains. In this section, we lay out some such criteria and discuss their application.

Identifying TE-derived lncRNA domains will be challenging for a number of reasons, not least the vast number of these sequences in the genome and the difficulty of using evolutionary filters on lineage-specific TE insertions. First, it is likely that many, if not the majority of exapted TE sequences will accumulate sequence changes such that we cannot identify them as repeat-derived sequence. A good example of this is the case of XIST, where the A-repeats are not annotated as having a TE origin by RepeatMasker, but nevertheless a more focused study using BLAST showed them to derive from endogenous retrovirus (Elisaphenko et al. 2008). Thus, these studies are likely to have poor sensitivity for genuine exapted TEs.

It is important to note that the proportion of TEs extant in the genome that have function is unknown. Therefore, we must consider the possibility that genome-scale catalogs of TE-derived lncRNAs may include large numbers, and possibly a majority, of nonfunctional sequences. That is, the majority of TE exonic insertions may not contribute a beneficial change to lncRNA activity, and their sequence will either evolve under random drift (for neutral or weakly deleterious insertions) or be eliminated from the population (strongly deleterious insertions). Therefore, we must consider it likely that genome-scale catalogs of TE-derived lncRNA will be dominated by nonfunctional sequences under neutral evolution, and the hallmarks of functionality indicated below will have relatively weak signals. This effect will correlate with the evolutionary age of the TE family: More recently, transposed repeats will be less likely to have acquired function and will have a smaller fraction of functional members.

If exapted TEs come under purifying selection, then we may make another prediction that the signal from most of the genomic filters described in the following section should become more pronounced for each TE family as a function of the time since that family was active; in other words, we expect to have the most power to identify signatures of exaptation in older TEs, as the difference between neutral, nonfunctional instances compared to exapted instances becomes more pronounced. Unfortunately, these same cases may be the hardest to identify as being repeat derived due to their age, as mentioned above.

We must also be careful how to interpret evidence of evolutionary selection: Such selection may be acting on a DNA or RNA phenotype. Specifically, a TE sequence may be conserved because it is acting through DNA, perhaps as a transcription factor binding site (Johnson et al. 2006), and its transcription within a lncRNA is purely coincidental. With these caveats in mind, we here discuss criteria for the genome-wide discovery of candidate RIDL elements.

### Base-level overrepresentation

Providing a large proportion of a particular repeat family have been exapted, their sequence may be overrepresented as a fraction of lncRNA exonic sequence compared to genomic sequence as a whole. This has been observed for multiple TE families, whose sequences are strongly and statistically significantly enriched in lncRNA exons, particularly various classes of endogenous retroviruses (HERV, MLT, LTR) (Kelley and Rinn 2012). Perhaps surprisingly, other classes of TE were also found to be significantly underrepresented in lncRNA exons, including various Alu subtypes; this effect may equally result from TE functionality since potent TE fragments may be selected against in many lncRNA hosts, where their presence is somehow detrimental or inappropriate to function, and only maintained in a subset, where they confer a selective advantage. This is consistent with the various documented activities of Alu sequence, both in isolation and in lncRNA contexts (Yakovchuk et al. 2009; Gong and Maquat 2011). Thus, counterintuitively, we may also include underrepresentation as a potential signature of TE exaptation.

### TE subregion overrepresentation in lncRNA

**FIGURE 5.**
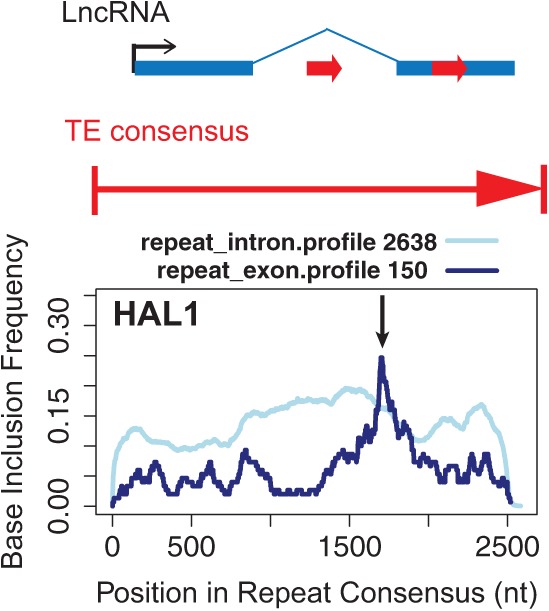
An approach to search for functional TE modules through insertion profiles. Preliminary data are shown for the LINE1-related HAL1 repeat. *Top*: a hypothetical lncRNA, containing TE fragment insertions in exonic and intronic regions (red arrows). *Bottom*: The plot shows base-level insertion frequency (*y*-axis), i.e., the probability of a given nucleotide being found in inserted fragments, with respect to position within the HAL1 consensus sequence (*x*-axis). Light blue and dark blue lines denote intronic and exonic data, respectively. The number of distinct insertion events upon which the data are based is shown *above* the plot.

TE families are comprised of a consensus motif that contains distinct subregions that have distinct sequence, structural, or functional properties (e.g., the UTRs and two ORFs of the LINE1 element) (Gifford et al. 2013; Goodier et al. 2013). Additionally, TEs tend to not insert their whole sequence during a novel insertion but rather insert a subfragment of their consensus motif, often variable lengths originating at the 3′ end due to incomplete reverse transcription (Lowe et al. 2007). We might expect that if particular subregions become exapted following insertion, then they will be overrepresented in the exons of host lncRNA, meaning that the frequency of observing particular fragments of a TE within lncRNA exons may differ from the genome as a whole. In Figure 5, we show preliminary data from our group, demonstrating the inclusion profile of the LINE1-like repeat, HAL1. The base-level inclusion profile in lncRNA exons is distinct from that of introns due to the presence of a peak of insertion specific to elements found in exons corresponding to a position around 1700 nt within the HAL1 consensus (indicated by an arrow), lying in the ORF region.

Such analysis of insertion profiles may be a useful method to filter functional lncRNA domains originating from TEs, although care should be taken in interpreting nonrandom profiles originating from processes such as exonization, which cannot be assumed to be indicative of function a priori. Once overrepresented TE subregions are found in lncRNA exons, the function of those regions in their endogenous TE transcript may hold clues to their role in the lncRNA. We predict that the most pronounced insertion profiles will reflect structures or protein-binding domains within TEs (Type I RIDLs) due to their specificity and relatively localized nature. In contrast, insertion profiles of Type II RIDLs will be expected to correspond to the profiles of their genomic or transcriptomic homology sites.

### Strand bias

If the function of a TE motif depends on the strandedness in which it is transcribed, then exapted TEs should preferentially be retained with a particular strand orientation relative to the host lncRNA exon. We have identified numerous cases of such strand bias for families of exonic TEs (R Johnson and R Guigó, unpubl.). A crucial consideration in these cases is that extreme strand bias will also be observed where TEs are contributing to lncRNA gene structures (Kapusta et al. 2013): Splice sites, promoters, or entire exons contributed by TEs will almost always occur through an element on one specific strand of the TE consensus, and therefore the resulting exonic TE regions will have a consistent strandedness with respect to the host transcript.

### Evolutionary conservation

Conservation is possibly the most powerful argument that can be used for function. Functional TE fragments should in principle display distinct evolutionary rates compared to similar fragments outside lncRNA exons that are assumed to be nonfunctional. Such a signature of selection was reported by Kapusta et al. (2013). However, this analysis was flawed since they specifically filtered intronic TE sequence to remove potential functional sequence that overlapped active chromatin marks without performing the same filtering on exonic TE sequences to which they were compared. Indeed, manual inspection reveals many instances of evolutionary conservation of TEs within lncRNA that in fact overlap genomic regulatory sites, i.e., the conservation is likely to arise as a result of DNA function of the sequence rather than RNA function, as has been observed previously (Lowe et al. 2007). This means that equal filtering of both exonic and intronic TEs must be carried out for such analyses to correctly understand the source of sequence conservation (either DNA or RNA function). Our unpublished global comparison of PhyloP base-level conservation of exonic and intronic sequence across all TE families does not reveal a significant signal of selection (R Johnson and R Guigó, unpubl.).

**FIGURE 6.**
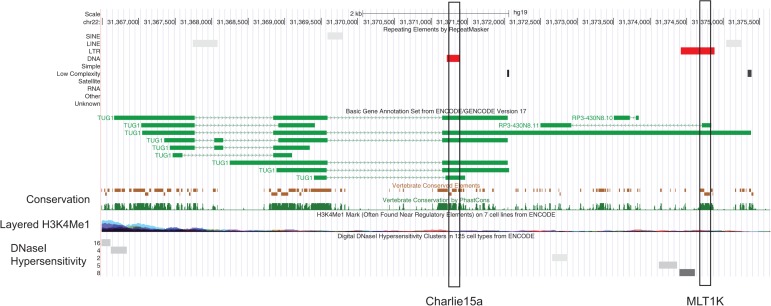
Map of the TUG1 lncRNA locus. Evolutionarily conserved candidates are indicated by red rectangles. PhastCons analysis of evolutionary conservation across vertebrates is shown in addition to histone modification and DNaseI hypersensitivity data from ENCODE. The absence of these signals in the two indicated TE insertions argues against evolutionary selection acting here on a DNA-encoded function.

However, this is not to say that individual repeat families may not have evolutionarily conserved sequence in exons. In support of this, there are many cases of apparent conservation of candidate RIDL sequences. By analyzing evolutionary conservation at each TE type in turn, we can find numerous cases with very strong evidence for purifying selection (Pollard et al. 2010; R Johnson and R Guigó, unpubl.). One example is shown in Figure 6, in which exons of the TUG1 transcript contain at least two evolutionarily conserved regions originating from Charlie15a and MLT1K transposons. Importantly, there is no evidence that these repeats function at the DNA level as revealed by absence of evidence of DNaseI hypersensitivity or chromatin modifications, consistent with the hypothesis that the evolutionary selection is here acting on an RNA-based phenotype.

Finally, an important consideration in the analysis of evolutionary conservation patterns on lncRNA will be exactly what is being conserved: sequence or structure? Most analyses of genomic conservation use sequence conservation, which likely has poor sensitivity in detecting the conservation of RNA structures. In contrast, a number of methods to specifically detect patterns of conservation in RNA structure have been presented, with increasing sensitivity (Washietl et al. 2005; Pedersen et al. 2006; Smith et al. 2013). It may be possible to take advantage of these differences in evolutionary forces to not only find evidence for selection but also to predict the function of repeat. Specifically, we predict that exapted TEs that work at the structural level (Type I) should display signals of conservation using methods adapted to RNA structure evolution such as ECS (Smith et al. 2013), whereas Type II TEs that depend on hybridization will be detected by more standard filters for purifying sequence selection such as PhastCons or PhyloP (Siepel et al. 2005; Pollard et al. 2010).

### Secondary structures

Exapted TE sequences may contain secondary structures that mediate their activity, and this may be reflected in a statistical overrepresentation of structured sequence. Many TEs are known to be highly structured, including Alu (Mariner et al. 2008). A simple metric, such as nucleotide-level propensity for base-pairing, could be used to search for statistical enrichment.

### Combinatorics

Recurring combinations of TEs may be apparent in lncRNA at nonrandom frequencies. Such combinations are observed in proteins, for example, in the frequent combination of KRAB-box repressor domains with zinc finger DNA-binding modules (Huntley et al. 2006). A possible example of this was mentioned previously in the context of XIST (Fig. 4A).

### Cellular localization

Some functional TE domains have been shown to associate with particular cellular compartments. For example, the SINEB2 domain of the Uchl1-as transcript regulates localization to the ribosome (Carrieri et al. 2012), or the Alu domain of ANRIL with chromatin (Holdt et al. 2013). Furthermore, TE RNAs in isolation tend to localize at different sites within the cell, e.g., SVA in the cytoplasm and Alu in the nucleus (Goodier et al. 2010) and the signal driving this localization presumably would act on lncRNA hosting those same TEs. Similarly, the analysis of subcellular RNAseq data may reveal enrichments of these and other TEs that could be acting as localization signals for lncRNA or else point to binding to other molecules with specific localization (Derrien et al. 2012; Djebali et al. 2012).

### Protein interaction

If TE sequences confer protein interaction domains on lncRNA, then we might expect to find signatures of this in experimental data sets of protein-TE interactions. The most obvious approach might be to search data sets such as recently published whole-genome maps of protein-RNA interactions represented by iCLIP or the related PAR-CLIP (Ule et al. 2003). One would expect to find protein-RNA interaction sites overlapping TE-derived fragments within lncRNA at higher than expected frequencies. A complementary approach, recently published by Lunyak's group, would be to experimentally catalog the protein-interactome of a given TE RNA. Here, the authors used Alu RNA as bait to identify the full set of interacting partners, finding a large number of DNA repair and epigenetic proteins (Blackwell et al. 2012). We might expect that such interactions are also retained by Alu fragments that occur within lncRNA, raising the possibility that Alu elements may form docking sites to chromatin proteins for lncRNA.

## OUTLOOK

In this review, we have argued that transposable elements represent a fundamental and versatile source of novel functional domains that facilitate the evolution of lncRNA. If this is correct, then the identification and characterization of these will represent a breakthrough in our ability to predict and manipulate functional lncRNA. A small but compelling set of examples attest to this, among them the functionally validated lncRNAs, XIST, ANRIL, RoR, and Uchl1-as. The demonstration that two distinct functional modules of XIST, the intensively studied and indispensable mammalian X-chromosome inactivation lncRNA, represent a powerful clue that such a mechanism may be widespread in lncRNA evolution. In addition to piecemeal identification of exapted TEs, we present a framework for genome-level identification of candidates. Hopefully, these data will eventually be integrated into methods that can accurately infer the activity of lncRNA based on their sequence alone.

## NOTE ADDED IN PROOF

While this paper was in press, another case of Alu-mediated DNA targeting of a lncRNA was published by Anindya Dutta and colleagues (Negishi et al. 2014).
